# Systemic Epstein–Barr Virus-Positive T/NK Lymphoproliferative Diseases With *SH2D1A*/*XIAP* Hypomorphic Gene Variants

**DOI:** 10.3389/fped.2019.00183

**Published:** 2019-05-21

**Authors:** Masataka Ishimura, Katsuhide Eguchi, Akira Shiraishi, Motoshi Sonoda, Yoshihiro Azuma, Hiroyuki Yamamoto, Ken-ichi Imadome, Shouichi Ohga

**Affiliations:** ^1^Department of Pediatrics, Graduate School of Medical Sciences, Kyushu University, Fukuoka, Japan; ^2^Department of Pediatrics, Yamaguchi University, Ube, Japan; ^3^Division of Advanced Medicine for Virus Infections, National Research Institute for Child Health and Development, Tokyo, Japan

**Keywords:** Epstein–Barr virus, chronic active EBV infection, lymphoproliferative disease, hemophagocytic lymphohistiocytosis, SAP, XIAP, X-linked lymphoproliferative disease

## Abstract

X-linked lymphoproliferative disease (XLP) is one of the X-linked primary immunodeficiency diseases (PIDs) with defective immune response to Epstein–Barr virus (EBV) infection. Chronic active EBV infection (CAEBV) and EBV-hemophagocytic lymphohistiocytosis (HLH) are recognized as systemic EBV-positive T-cell and natural killer (NK)-cell lymphoproliferative diseases (LPDs) arising from the clonal proliferations of EBV-infected T cells and NK cells. A high incidence of CAEBV in East Asia implies the unknown genetic predisposition. In patients with XLP, EBV-infected cells are generally B cells. No mutation of *SH2D1A*/*XIAP* genes has ever been identified in patients with systemic EBV-positive T-cell and NK-cell LPD. We report herewith a male case of NK-cell type CAEBV with *SH2D1A* hypomorphic mutation (c.7G > T, p.Ala3Ser), two male cases of CAEBV/EBV-HLH with *XIAP* hypomorphic variant (c.1045_1047delGAG, p.Glu349del), and another female case of CD4^+^CAEBV with the same *XIAP* variant. The female underwent bone marrow transplantation from an HLA-matched sister with the *XIAP* variant and obtained a complete donor chimerism and a cure of laryngeal LPD lesion, but then suffered from donor-derived CD4^+^ T cell EBV-LPD. These observations demonstrated that *SH2D1A* and *XIAP* genes are critical for the complete regulation of EBV-positive T/NK cell LPD. X-linked lymphoproliferative disease (XLP) is one of the X-linked primary immunodeficiency diseases (PIDs) reported to have a defective immune response to Epstein–Barr virus (EBV) infection. Mutations in *SH2D1A* and *XIAP* genes cause XLP. Systemic EBV-positive T-cell and natural killer (NK)-cell lymphoproliferative diseases (LPDs) consist of three major types: EBV-positive hemophagocytic lymphohistiocytosis (HLH), chronic active EBV infection (CAEBV), and EBV-positive T-cell/NK-cell lymphoma. CAEBV is recognized as a poor prognostic disease of EBV-associated T-cell and NK-cell LPD arising from the clonal proliferation of EBV-infected T cells (CD4^+^, CD8^+^, and TCRγδ^+^) and/or NK cells. The majority of cases with CAEBV were reported from East Asia and South America. In Caucasian patients with CAEBV disease, the target of infection is exclusively B cells. These imply a genetic predisposition to EBV-positive T/NK cell LPD according to ethnicity. In reported cases with XLP, EBV-infected cells are B cells. On the other hand, no mutation of *SH2D1A*/*XIAP* genes have been determined in patients with T/NK-cell-type (Asian type) CAEBV. We here describe, for the first time, four case series of CAEBV/EBV-HLH patients who carried the hypomorphic variants of XLP-related genes. These cases included a male patient with CAEBV carrying *SH2D1A* hypomorphic mutation (c.7G > T, p.Ala3Ser) and two male patients with CAEBV/EBV-HLH carrying the *XIAP* hypomorphic variant (c.1045_1047delGAG, p.Glu349del), along with another female patient with CAEBV carrying the same *XIAP* variant. The female case underwent bone marrow transplantation from a healthy HLA-matched sister having the same *XIAP* variant. Although a complete donor chimerism was achieved with the resolution of laryngeal LPD lesions, systemic donor-derived CD4^+^ T-cell EBV-LPD developed during the control phase of intractable graft- vs. -host-disease. These observations demonstrated that *SH2D1A* and *XIAP* genes are critical for the complete regulation of systemic EBV-positive T/NK-cell LPD.

## Introduction

Epstein–Barr virus (EBV) infects preferentially human B lymphocytes and epithelial cells. The majority of subjects are asymptomatic after a primary infection with EBV, and a part of them suffer from acute infectious mononucleosis (IM). EBV-positive T-cell and natural killer (NK)-cell lymphoproliferative diseases (LPDs) are classified into three major types: EBV-positive hemophagocytic lymphohistiocytosis (HLH), chronic active EBV infection (CAEBV), and EBV-positive T-cell/NK-cell lymphoma (1). CAEBV is a rare persistent active mononucleosis syndrome presenting with fever, liver dysfunction, cytopenias, and hepatoplenomegaly. The patients occasionally progress to the lethal course of HLH, LPD, and lymphoma. CAEBV is currently recognized as EBV-associated T/NK-cell LPD arising from the clonal proliferations of EBV-infected T cells (CD4^+^, CD8^+^, and TCRγδ^+^) and/or NK cells. The majority of cases were reported from East Asia and South America. The reported cases of chronic EBV disease in the United States were exclusively B-cell-type (Caucasian type) CAEBV (2). These may account for the genetic predisposition to T/NK-cell-type (Asian type) CAEBV (3). Recently, Okuno et al. (4) have reported that somatic mutations of infected cells and gene mutations of EBV were concurrently involved in the development of T/NK cell type (Asian type) CAEBV, which were considered to be similar to malignant lymphoma. Allogenic hematopoietic stem cell plantations (HSTs) are needed for curing of progressive CAEBV (5). On the other hand, there is little information on the prolonged survival of patients with indolent CAEBV.

X-linked lymphoproliferative disease (XLP) is one of the X-linked primary immunodeficiency diseases (PIDs) with defective immune response to EBV infection (6). The manifestations of XLP are typified by EBV-driven fatal IM and/or HLH, regenerative anemia, dysgammaglobulinemia, and B-cell lymphoma. In cases of XLP-related EBV-HLH, EBV-infected cells are predominantly B cells (7, 8). Currently, two causative genes have been determined for XLP1 and XLP2: SLAM-associated protein (SAP) deficiency due to *SH2D1A* gene mutation called XLP1 and XIAP (X-linked inhibitor of apoptosis) deficiency due to *XIAP* gene (previously termed *BIRC4*) mutation called XLP2. SAP is composed almost exclusively of an Src homology 2 (SH2) domain (9). SAP expressed in T cells, NK cells, and natural killer T (NKT) cells enhances immune response binding to signaling lymphocyte activation molecule (SLAM) families. In XLP1 patients, decreased cytotoxic activities in CD8^+^ T cells and NK cells are associated with the developing risk of HLH. In addition, the lack of invariant NKT (iNKT) cells leads to a nonexcludability of EBV-infected B cells and subsequent development of fatal IM or B-cell lymphoma. XIAP is a member of the inhibitor of apoptosis protein (IAP) family containing three baculovirus IAP repeat (BIR) domains and one really interesting new gene (RING) domain. XIAP regulates apoptotic cell death with the inhibition of procaspase 9 by BIR3 domain and the inhibition of caspase 3 and 7 by BIR2 domain (10). The RING domain ubiquitylates receptor-interacting serine/threonine-protein kinase 2 and recruits linear ubiquitin chain assembly complex (LUBAC) to nucleotide-binding oligomerization domain 2 (NOD2). LUBAC activity is required for efficient NF-κB activation and secretion of proinflammatory cytokines after NOD2 stimulation (11). Increased activation-induced cell death (AICD) in T cells and a decreased number of iNKT cells are found in XIAP-deficient patients (12). XLP2 patients with a mutation in the RING domain exhibit interference with ubiquitin ligase activity (11). The symptoms start usually in early childhood with recurrent infection, with HLH (frequently recurrent and of a more indolent course than seen in other primary HLH diseases) associated with chronic EBV disease, splenomegaly, and chronic colitis. In contrast to XLP1, B-cell lymphomas have not been reported in XLP2 patients (13).

We herein describe four novel cases of systemic EBV-related T/NK-LPD having the hypomorphic variants of *SH2D1A*/*XIAP* and discuss their association.

## Methods

### Genetic Analysis

Genomic DNA was extracted from peripheral blood and/or biopsied samples of the lesion obtained from patients according to the standard method, after informed consent was obtained from the individuals or parents. Mutation analysis of the genes responsible for familial HLH (*PRF1, UNC13D, STX11*, and S*TXBP2*) and XLP or XLP-like (*SH2D1A, XIAP, ITK*, and *CD27*) was performed by a PCR-assisted DNA sequencing method with a capillary DNA sequencer (ABI3130/3730, Applied Biosystems, Foster City, CA, USA) in all cases, and the whole exome sequencing, using a short-read next-generation DNA sequencer (MiSeq, Illumina, San Diego, CA, USA) as described previously (14) in case 1 and case 4.

### EBV Analysis

Analysis of EBV gene expression by real time-PCR was carried out as previously described (15). Briefly, peripheral blood mononuclear cells (PBMCs) were isolated by centrifugation on a Lymphosepar I (Immuno-Biological Laboratories Co., Ltd., Gunma, Japan). Then, CD19^+^, CD4^+^, CD8^+^, and CD56^+^ cells were serially removed from PBMCs using the IMag Cell Separation System (BD Biosciences, Franklin Lakes, NJ, USA). DNA extraction was performed for each fraction, and quantification of EBV-DNA was performed with real-time quantitative PCR based on the TaqMan system (Applied Biosystems, Foster City, CA, USA).

## Clinical Case Reports

### Case 1: Male Patient With NK-Cell-Type CAEBV

An 8-years-old boy presented with high fever, photosensitivity, and hypersensitivity to mosquito bites and then received the diagnosis of NK-cell-type CAEBV. These manifestations have gradually relieved until 12 years of age. The comprehensive genetic analysis of peripheral blood-derived DNA revealed one reported pathological mutation of *SH2D1A* gene hemizygously (c. 7G > T, p.Ala3Ser) (16, 17). During the following 13 years, he has continued to have photosensitivity alone. Repeated laboratory tests have shown unremarkable titers of anti-EBV antibodies indicating past infection and low titer of EBV genome copies in peripheral blood (7.3 × 10^2^/ml), with no any evidence of cytopenia, dysgammagulobulinemia, or elevation in soluble interleukin (IL)-2 receptor.

### Case 2: Male Patient With NK/B-Cell-Type CAEBV

A 2-years-old boy had suffered from intermittent fever, diarrhea, and hypersensitivity to mosquito bites. An EBV genome load was high in CD19^+^ B cells (5.6 × 10^3^ copies/μgDNA) and slightly positive levels in CD16^+^ NK cells (8.1 × 10^1^ copies/μgDNA). The comprehensive genetic analysis of peripheral blood-derived DNA determined a reported hemizygous variant of *XIAP* gene (c.1045_1047delGAG, p.Glu349del) (7, 8). NK cell activity was 18 %lysis (reference range; 18–40). After the diagnosis of chronic EBV^+^B-LPD, four courses of anti-CD20 antibody (Rituxan®, Chugai Pharmaceutical Co., LTD., Tokyo, Japan) therapies led to a complete disappearance of the EBV genome in circulation and an improvement in hypersensitivity to mosquito bites. Six months after rituximab therapies, a reappearance of B cells in the peripheral blood without the detection of EBV genome indicated the eradication of EBV-B-LPD. However, EBV genome level was again positive (1.5 × 10^3^ copies/μgDNA of whole peripheral blood) 10 months after rituximab therapy, but there were no symptoms or abnormal data, including immunoglobulin levels, in the follow-up screening tests.

### Case 3: Male Patient With CD8^+^ T-Cell-Type EBV-HLH

A 1-year-old boy developed fever, skin eruptions, and hepatoplenomegaly with pancytopenia, hyperferritinemia (5,181 ng/ml), and elevated soluble IL-2 receptor (6,797 U/ml). Anti-EBV antibodies indicated a primary infection of EBV. High EBV loads in peripheral blood and CD8^+^ T cells of the patient (1 × 10^5^ copies/ml and 1 × 10^6^ copies/μgDNA, respectively) led to the diagnosis of EBV-HLH. NK-cell activity was 30 %lysis in normal (reference range; 18–40). Additional two courses of etoposide injection (100 mg/m^2^) were needed to control the relapsing HLH after the immunomodulation therapy using high-dose intravenous immunoglobulin, oral cyclosporine, and prednisolone. Circulating levels of EBV genome came to be undetectable after the immunochemotherapy. The comprehensive genetic analysis of peripheral blood-derived DNA determined a hemizygous variant of the *XIAP* gene (c.1045_1047delGAG, p.Glu349del). He is alive and well, without sequelae or dysgammaglobulinemia at 7 years of age. The numbers of CD19^+^IgD^−^CD27^+^ switched memory B cells and CD4^+^CD45RA^−^CXCR5^+^ follicular helper T cells were not decreased (data not shown).

### Case 4: Female Patient With NK/CD4^+^ T-Cell-Type CAEBV

**Figure 1:**
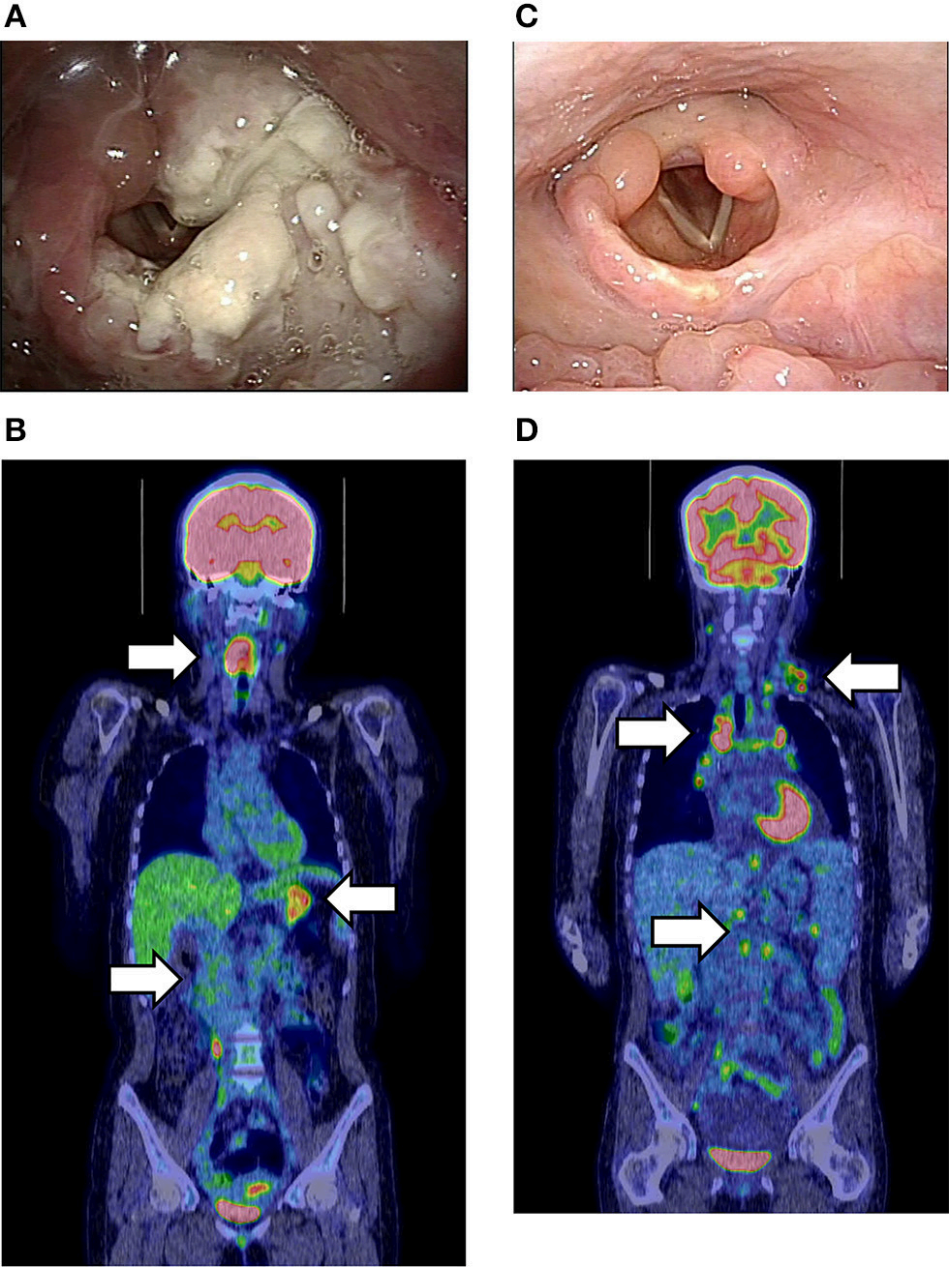
**(A)** Endoscopic findings of the laryngeal lesion of CD4^+^T-cell lymphoproliferative disease (LPD) lesion in case 4 prior to cancer chemotherapy. **(B)** Fluorodeoxyglucose-positron emission tomography (FDG-PET) at the onset of LPD. White arrows show the increased FDG uptake in the larynx, stomach, and terminal ileum. The maximum standardized uptake value (SUVmax) was 11.8. **(C)** Improvement of the laryngeal LPD lesion after bone marrow transplantation (BMT). **(D)** FDG-PET at the onset of donor-derived CD4+T-LPD after BMT. White arrows show the increased FDG uptake in multiple lymph nodes without laryngeal lesion. The SUVmax was 9.7.

A 24-years-old woman was hospitalized because of dyspnea and hoarseness. The patient had received the diagnosis of NK-cell and CD4^+^ T-cell-type CAEBV because of recurrent fever and hypersensitivity to mosquito bites at age 14 years. Histopathological and molecular studies of the cutaneous lesion indicated clonal proliferation of EBV-infected cells. Thereafter, clinical resolution and declining levels of EBV load in circulation had allowed no treatment and observation. After admission, an urgent tracheostomy prevented airway obstruction by the laryngeal mass (Figure 1A). She was then transferred to our hospital for further management. Fluorodeoxyglucose-positron emission tomography (FDG-PET) showed increased levels of uptake in the stomach and terminal ileum as well as the laryngeal lesion (Figure 1B). Circulating EBV DNA was at undetectable levels. However, histopathological and molecular analysis of the laryngeal lesions demonstrated a proliferation of EBER-positive CD4^+^ cells and increased copy number of EBV-DNA (2–4 × 10^3^ copies/μgDNA). The comprehensive genetic analysis of peripheral blood-derived DNA identified a heterozygous variant of *XIAP* gene (c.1045_1047delGAG, p.Glu349del) alone. A histocompatible sister aged 20 years carried the same *XIAP* variant. The anti-EBV antibody titers and undetectable EBV DNA in circulation verified a past infection of EBV in the healthy sister. The gene expression analysis indicated no skewing inactivation of X chromosome among DNA samples obtained from the bone marrow cells, PBMCs, and laryngeal tumor of the patient as well as PBMC of the sister (data not shown). After four courses of combined chemotherapies with cyclophosphamide, pirarubicin, vincristine, steroid, and etoposide (CHOP-VP), the patient underwent bone marrow transplantation from the sister. The laryngeal lesion disappeared after a compete donor chimerism was achieved (Figure 1C). However, systemic but not local proliferation of EBV-infected donor-derived CD4^+^ T cells (1 × 10^4^ copies/ml of whole peripheral blood and 3 × 10^3^ copies/μgDNA of CD4^+^T cells, respectively) developed 2 months posttransplantation (Figure 1D). Discontinuation of immunosuppresants and donor lymphocyte infusions effectively controlled the posttransplantation LPD, but she died of uncontrollable severe graft-vs.-host-disease with Candida sepsis.

None of the four patients had a positive family history suggesting PID and/or chronic EBV disease. The clinical profile and treatment course of these patients are summarized in Table 1.

**Table 1:** Summary of the patients with *SH2D1A*/*XIAP* mutations who developed EBV-infected T/NK cell LPD.

**Case**	**Sex**	**Gene mutation**	**Diagnosis**	**Age at onset (years)**	**Age at treatment (years)**	**EBV-infected cell type**	**Treatment**	**Follow-up period (years)**	**Outcome**
1	Male	*SH2D1A*: c.7G>T (hemizygous)	CAEBV	8	Not yet	NK cells	none	17	Alive
2	Male	*XIAP*: c.1045_1047delGAG (hemizygous)	CAEBV	2	2	B cells and NK cells	RTX (four courses)	2	Alive
3	Male	*XIAP*: c.1045_1047delGAG (hemizygous)	EBV-HLH	1	1	CD8^+^T cells	VP-16, PSL, IVIG	7	Alive
4	Female	*XIAP*: c.1045_1047delGAG (heterozygous)	CAEBV	14	24	CD4^+^T cells	Chemotherapy^*^ (four courses), rBMT	12	Deceased

CAEBV, chronic active Epstein-Barr virus; EBV, Epstein-Barr virus; EBV-HLH, EBV-associated hemophagocytic lymphohistiocytosis; IVIG, intravenous immunoglobulin; LPD, lymphoproliferative disorder; PSL, prednisolone; rBMT, related donor bone marrow transplantation; RTX, rituximab.

* *Chemotherapy: etoposide 100–150 mg/m^2^ × 3 days, cyclophosphamide 750 mg/m^2^ × 1 day, vincristine 1.5 mg/m^2^ × 1 day, pirarubicin 25 mg/m^2^ × 2 days, PSL 1 mg/kg × 3 days*.

## Discussion

We report the first case series of CAEBV/EBV-HLH patients who carried the hypomorphic mutation of XLP-related genes. B cells are the major target of EBV infection in patients with XLP. No mutation in *SH2D1A*/*XIAP* genes has been reported in patients with CAEBV (4, 18). Patient 1 with SAP Ala3Ser and patients 2, 3, and 4 with XIAP Glu349del presented with the phenotype of NK^+^CAEBV, B^+^/NK^+^CAEBV, CD8^+^EBV-HLH, and NK^+^/CD4^+^CAEBV, respectively. EBV preferentially infects B cells *via* CD21 and also infects T cells or NK cells at a lower frequency during the acute phase of viremia (3). The absolute number of EBV-infected T/NK cells would be too small to have an advantage for proliferating in healthy individuals. On the other hand, SAP/XIAP variant carriers may have a modest ability to control the proliferation of EBV-infected T/NK cells.

Low levels of circulating EBV DNA continue to be detected in NK cells, but no symptoms other than photosensitivity have continued in patient 1 for 17 years. The *SH2D1A* gene variant (c.7G > T, p.Ala3Ser) is located at the N-terminal of the SAP protein, outside of the SH2 domain, with residual protein functions. This variant has no deleterious effect on the function depending on SH2 domain. A healthy elderly male carrier with c.7G > T *SH2D1A* variant indicates that the variant does not always develop XLP or lead to fatal outcomes for patients (17). However, one male patient with the same p.Ala3Ser variant demonstrated a marked reduction in SAP expression in CD8^+^ T cells (2.7%, reference range: 21.6–90.8%) and died at the age of 40 years with EBV infection and HLH (16). Another male patient with the hemizygous *SH2D1A* variant (c.7G > T) and heterozygous missense *PRF1* mutation (c.127C > A, p.Leu43Met) reportedly suffered from severe HLH and required HST, and the other female with the heterozygous *SH2D1A* variant (c.7G > T) had a lethal HLH (19). The *SH2D1A* c.7G > T may be pathogenic in cases with late onset or indolent expression of disease.

The XIAP Glu349del (rs199683465) was reportedly detected in 3.5% of healthy Japanese people. The variant may be a single nucleotide polymorphism exerting the founder effect in Japanese people (20). Although the XIAP protein expression was normal in the Glu349del variant, the numbers of CD19^+^IgD^−^CD27^+^ switched memory B cells and CD4^+^CD45RA^−^CXCR5^+^ follicular helper T cells were decreased and immunoglobulin production was reduced *in vitro*. Immunoglobulin-related gene expression was also decreased in the variant carriers. On the other hand, they did not exhibit increased AICD because Glu349 is distant from the BIR2 or BIR3 domains. It remains unclear how the Glu349del mutation affects the NOD pathways (20).

Male patient 2, with persistent EBV infection in B/NK cells accompanied by hypersensitivity to mosquito bites, was diagnosed as having CAEBV. Rituximab therapy temporarily cleared EBV genome and all signs and symptoms. Although the EBV copy number was increased again, no symptoms recurred. Persistent EBV infection leads to the diagnosis of XLP2 without dysgammaglobulinemia or HLH. Patient 3 is the first Japanese individual who is a Glu349del carrier who developed EBV-HLH. No HLH developed in the three reported Japanese XLP2 patients with Glu349del (20). On the other hand, a 1-year-old Chinese male with XIAP Glu349del has been recently reported to present with HLH (21). Patient 3 suffered from severe CD8^+^EBV-HLH requiring chemotherapy, which did not recur after the first resolution. Unlike other *XIAP* mutations, the Glu349del variant would not lead to uncontrollable or relapsing HLH.

The major concern is the intractable course of patient 4 posttransplantation. How did a heterozygous *XIAP* variant affect the female patient? The *XIAP* variant is a genetic polymorphism found in 3.5% of healthy Japanese individuals. The healthy sister of patient 4 had experienced a primary infection of EBV. Therefore, heterozygous variant carriers are less likely to have acute fatal IM and/or chronic EBV diseases. X-linked recessive PID develops in females with X chromosome skewing (22, 23), but no skewing expression of *XIAP* was determined in the PBMC or LPD lesion of patient 4. EBV DNA levels did not increase in the peripheral blood even at the time of developing LPD after 10 years from the first CAEBV diagnosis. Additional factors such as somatic mutations and EBV genome mutations might contribute to the evolution of laryngeal tumor (4). Furthermore, as shown from her developing posttransplanted donor-derived EBV-related CD4^+^T-LPD, unknown host genetic predispositions other than *XIAP* or immunocompromised state of suppressing EBV-specific CTL activity might be involved in the onset of CAEBV (24). Somatic mutations of EBV-infected donor CD4^+^ T cells might also affect the developing posttransplantation EBV-T-LPD (4). Genetic backgrounds, including hypomorphic variants, may need to be considered in the selection of HST donors for the cure of systemic EBV-positive T/NK cell LPD.

## Conclusions

We reported the first four case series of CAEBV/EBV-HLH with *SH2D1A*/*XIAP* gene variants. PID-related genetic predispositions to EBV infection should be considered for the treatment of EBV-T/NK cell LPD.

## Ethics Statement

This study was carried out in accordance with the Declaration of Helsinki and the recommendations of the institutional review board of Kyushu University. The protocol was approved by the institutional review board of Kyushu University (#531-01). The subjects (or their parents) gave written informed consent about the study and the publication of this report.

## Author Contributions

MI and SO were the principal investigators, taking primary responsibility for the paper. AS, KE, MS, and YA performed the clinical management with helpful discussion regarding the completion of the work. KI completed the EBV analysis. MS and HY completed the genetic analysis.

### Conflict of Interest Statement

The authors declare that the research was conducted in the absence of any commercial or financial relationships that could be construed as a potential conflict of interest.

## Abbreviations

AICD: activation-induced cell death
BIR: baculovirus IAP repeat
BMT: bone marrow transplantation
CAEBV: chronic active Epstein–Barr virus infection
EBV: Epstein–Barr virus
FDG-PET: fluorodeoxyglucose-positron emission tomography
HLA: human leukocyte antigens
HST: hematopoietic stem cell transplantation
HLH: hemophagocytic lymphohistiocytosis
IL: interleukin
LPD: lymphoproliferative disease
LUBAC: recruits linear ubiquitin chain assembly complex
NK: natural killer
NOD2: nucleotide-binding oligomerization domain 2
PID: primary immunodeficiency disease
RING: really interesting new gene
SAP: SLAM-associated protein
SH2: Src homology 2
SNP: single nucleotide polymorphism
XIAP: X-linked inhibitor of apoptosis
XLP: X-linked lymphoproliferative disease.
